# Machine Learning–Enabled Clinical Information Systems Using Fast Healthcare Interoperability Resources Data Standards: Scoping Review

**DOI:** 10.2196/48297

**Published:** 2023-08-24

**Authors:** Jeremy A Balch, Matthew M Ruppert, Tyler J Loftus, Ziyuan Guan, Yuanfang Ren, Gilbert R Upchurch, Tezcan Ozrazgat-Baslanti, Parisa Rashidi, Azra Bihorac

**Affiliations:** 1 Department of Surgery, University of Florida Health, Gainesville, FL, United States; 2 Intelligent Critical Care Center, University of Florida, Gainesville, FL, United States; 3 Department of Medicine, University of Florida, Gainesville, FL, United States; 4 Department of Biomedical Engineering, University of Florida, Gainesville, FL, United States

**Keywords:** ontologies, clinical decision support system, Fast Healthcare Interoperability Resources, FHIR, machine learning, ontology, interoperability, interoperable, decision support, information systems, review methodology, review methods, scoping review, clinical informatics

## Abstract

**Background:**

Machine learning–enabled clinical information systems (ML-CISs) have the potential to drive health care delivery and research. The Fast Healthcare Interoperability Resources (FHIR) data standard has been increasingly applied in developing these systems. However, methods for applying FHIR to ML-CISs are variable.

**Objective:**

This study evaluates and compares the functionalities, strengths, and weaknesses of existing systems and proposes guidelines for optimizing future work with ML-CISs.

**Methods:**

Embase, PubMed, and Web of Science were searched for articles describing machine learning systems that were used for clinical data analytics or decision support in compliance with FHIR standards. Information regarding each system’s functionality, data sources, formats, security, performance, resource requirements, scalability, strengths, and limitations was compared across systems.

**Results:**

A total of 39 articles describing FHIR-based ML-CISs were divided into the following three categories according to their primary focus: clinical decision support systems (n=18), data management and analytic platforms (n=10), or auxiliary modules and application programming interfaces (n=11). Model strengths included novel use of cloud systems, Bayesian networks, visualization strategies, and techniques for translating unstructured or free-text data to FHIR frameworks. Many intelligent systems lacked electronic health record interoperability and externally validated evidence of clinical efficacy.

**Conclusions:**

Shortcomings in current ML-CISs can be addressed by incorporating modular and interoperable data management, analytic platforms, secure interinstitutional data exchange, and application programming interfaces with adequate scalability to support both real-time and prospective clinical applications that use electronic health record platforms with diverse implementations.

## Introduction

Data analytic tools provide essential contributions to scientific investigation and clinical decision-making [1]. These tools are in turn fueled by the volumes of data that have been generated since the passage of the Health Information Technology for Economic and Clinical Health Act in 2009, which incentivized the adoption of electronic health record (EHR) systems [2-4].

EHR data, however, remain nonstandardized across institutions and, within an institution, may not be readily available for real-time analysis, thus impairing multi-institutional research efforts and care for individual patients across institutions [5-8]. The standards herein refer to the structure, organization, representation, and transmission of data. Health information exchange systems can mitigate these issues by using the Fast Healthcare Interoperability Resources (FHIR; pronounced “fire”) data standard [9]. The Health Level 7 (HL7) International standard developing organization sought to reduce the complexity of the HL7 version 3 Reference Information Model while maintaining semantic interoperability and thus adopted the FHIR standard in 2011 [10]. It supports multiple development platforms and has been embraced by major industry and government organizations. Since 2016, developers have engaged with Substitutable Medical Applications and Reusable Technologies (SMART) on FHIR to build EHR and commercial applications [11,12]. Despite the growth of technologies using FHIR standards, there is limited literature summarizing differences among machine learning–enabled clinical information systems (ML-CISs), and the best methods for applying FHIR remain unclear.

This review describes the functionalities, strengths, and weaknesses of clinical applications that use the FHIR standard and have been described in the medical literature, and we propose guidelines for improved multi-institutional research initiatives and clinical applicability.

## Methods

Given the rapidly evolving nature of this field, we performed a scoping review to provide a critical appraisal of the current literature, with the goal of informing future studies. We followed the PRISMA-ScR (Preferred Reporting Items for Systematic Reviews and Meta-Analyses extension for Scoping Reviews) guidelines; the PRISMA-ScR checklist is available in Multimedia Appendix 1.

### Research Protocol

We sought articles describing clinical decision support (CDS) systems (CDSSs) or risk prediction systems using FHIR standards. FHIR standards define resource types (ie, patients, medications, and clinical observations), data elements (ie, medication name and dosage), data formats (ie, JSON and XML files), and the use of standard ontologies (ie, Systematized Nomenclature of Medicine-Clinical Terms [SNOMED CT] and Logical Observation Identifiers Names and Codes [LOINC]), among others. Our initial search was performed on April 23, 2020, and given the progress of the field, it was updated again on October 11, 2022. Inclusion criteria involved all full-text articles published in English. We excluded abstracts, poster presentations, and meeting summaries. Embase, PubMed, and Web of Science were searched for cohort studies, case-control studies, and reviews. Our search terms for each database are found in Multimedia Appendix 2. Despite their increasing use by commercial entities, we did not search for commercial applications of FHIR, as their lack of peer review and limited reportability prevented a formal evaluation of their methods. Following the removal of duplicates, 153 articles were identified. Titles and abstracts were reviewed by 2 authors independently, with disagreements resolved by a third. Full-text articles that did not adequately describe system functionality, data sources, formats, security, performance, resource requirements, scalability, strengths, and limitations were excluded. We also excluded articles that described a model architecture using FHIR but did not incorporate it into a CDSS. A total of 39 full-text articles were included for full analysis.

### Article Evaluation

Strengths and limitations of the applications were evaluated in terms of functionality, data sources, formats, security, performance, resource requirements, and scalability. *Functionality* was defined as the intended purpose of the algorithm and its capabilities, ranging from the integration of genomic data into the EHR [13,14] to CDSSs [15-17] and predictive models [18,19]. Data sources included information within electronic health care records and external sources, such as wearable devices [20]. Formats were evaluated based on system architecture and the technologies underlying the algorithms (eg, use of Bayesian networks [16], transformers [21], or rule-based methods [22]). Security was evaluated based on how the application handled sensitive health information, including encryption [23], use-and-access control mechanisms [24], or authorization platforms [25,26]. Performance and resource requirements refer to the processing time, memory, and computing needs of the applications. Finally, scalability refers to the likelihood of adoption by other health care systems or platforms (eg, use of open-source components [27] or cloud-based repositories [28]). Knowledge from the included articles was used to propose avenues of future development for optimizing machine learning–enabled systems.

## Results

A total of 39 clinical tools that used FHIR standards were divided into the following three categories according to their primary focus: CDSSs (n=18), interoperable data management and analytic platforms (n=10), or auxiliary modules and application programming interfaces (APIs; n=11) that enhance ML-CISs.

### The CDSSs

CDSSs are algorithms that use health information to provide assistance for clinical decision-making tasks. Table 1 shows articles that focused on these support systems. Although many CDSSs lacked interoperability and external validity, several characteristics of CDSSs harbored potential for improving both efficacy and efficiency.

**Table 1.** Summary of intelligent clinical decision support systems.

Source, year	Functionalities	Strengths	Limitations
Curran et al [15], 2020	Summarizes chronic obstructive pulmonary disease information, provides decision support, and suggests orders	Dynamic embedding within EHR^a^ and compatible with SMART^b^-on-FHIR^c^ submodules	Limited generalizability due to single center and single disease
Dolin et al [13], 2018	Uses drug-gene interaction data for clinical decision support triggered by EHR medication orders	Accesses a rules engine containing level A recommendations from a pharmacogenetics consortium	Difficult to query the rules engine for level A recommendations when triggered by EHR medication orders
El-Sappagh et al [28], 2019	Uses mobile health technologies to monitor and manage type 1 diabetes	Most system processes are executed in the cloud; once configured, it runs on any EHR system	The diabetes treatment ontology did not address emergency conditions and was not embedded within an EHR system
Gaebel et al [16], 2016	Generates digital patient models for clinical decision support for laryngeal cancer	Bayesian networks are well-suited for representing complex diseases	System architecture was described, but the system was not implemented clinically
Gruender et al [14], 2019	Combines next-generation sequencing genomics data with FHIR clinical data	Open-source system that combines data formats and is portable	Manual data extraction and web-based filtering tool
Gordon et al [29], 2017	Displays patients’ thrombocytopenia trends along with computer-generated calculated panel reactive antibody levels	Provides real-time services and effective visual cues	Data sources are limited
Henry et al [18], 2018	Predicts sepsis among intensive care unit patients in real time	Cloud-based system that provides alerts to clinicians	Public cloud-based solutions present safety issues
Hong et al [27], 2019	Phenotypes diabetes based on free-text notes and other structured data	Converts unstructured, semistructured, and structured data to appropriate FHIR components	Performance is not stable across different data sets
Kawamoto et al [30], 2021	Takes data from multiple EHRs and incorporates them into existing risk calculators	Performance measured with end user satisfaction studies and used existing application programming interface	Tested at a single institution and had data security concerns
Park et al [31], 2022	Personal health record application for employees, with links to health care resources	FHIR-based cloud application that is applicable to multiple EHRs and provides secure access through Azure	Limited integration of hospital data
Schleyer et al [32], 2021	Integrates selected data from statewide data systems into local EHR	Translates data from diverse sources into a common database	Experience limited to a single EHR
Semenov and Kopanitsa [20], 2018	Recommends clinical decisions and actions based on EHR data	Free-text output for both physicians and patients	No standard performance evaluation
Semenov et al [33], 2018	Recommends clinical decisions and actions based on EHR data	Free-text output for both physicians and patients and improved analytic workflow relative to prior versions	No standard performance evaluation
Séroussi et al [34], 2018	Produces clinical practice guideline services for patients with breast cancer	Uses both data models and knowledge models and provides effective data analytic visualizations	Implemented on a small scale, proposed guidelines were not validated, and interguideline conflicts need to be resolved manually
Tarumi et al [35], 2021	Modeling of treatment outcomes for type 2 diabetes	Effective use of SMART on FHIR for integration in local EHR, and design incorporated clinician feedback	No external validation, limited access to cost data, and not yet compatible with all EHRs
Thayer et al [36], 2021	Automated graphical display of asthma history	Smoothly integrated into EHR	Not based on SMART, limiting interoperability
Wang et al [37], 2019	Comparison of machine learning algorithms for prediction of end-stage renal disease in type 2 diabetes	Extraction of EHR data using FHIR	Single institution, no imputation of missing data, and no external validation
Whitaker et al [38], 2022	Machine learning algorithm to identify blood transfusion adverse events	Synthesized structured and unstructured data from EHR to achieve reasonable accuracy compared to clinicians	Retrospective study more aligned toward research than clinical care

a EHR: electronic health record.

b SMART: Substitutable Medical Applications and Reusable Technologies.

c FHIR: Fast Healthcare Interoperability Resources.

CDSS ontologies are a central tenant of CDSS interoperability. Generally, ontologies are a hierarchy of concepts that are defined by both a set of attributes and their relationships to other concepts, and they must meet several internal consistency and version control objectives [10]. Common ontologies include the SNOMED CT, LOINC, and National Cancer Institute Thesaurus (NCIT). Separate ontologies may conflict, such as in cases where models use different organizing principles, have varying degrees of granularity, or even exhibit contextual differences between clinical applications and biomedical research. Séroussi et al [34] faced this problem when creating a guideline for the optimal management of breast cancer by integrating a collection of pre-existing ontologies (NCIT and LOINC). They were able to resolve this conflict by using data visualization techniques and rules-based inference engines, though often their methods required the manual resolution of conflicts. Common ontologies can also omit essential elements. Dolin et al [13] were able to transform a library of drug-gene interactions into an FHIR standard to alert physicians when prescriptions are likely to cause adverse drug reactions. Specific disease classes may lack an interoperable ontology. For cancer, there are active efforts in the CodeX HL7 FHIR Accelerator community to capture oncologic data from the EHR by using the mCODE (minimal Common Oncology Data Elements) ontology [39,40].

Advanced CDSSs have been integrated with machine learning algorithms to process data, especially unstructured data, such as clinician notes. Gaebel et al [16] created a physician-facing CDSS that used Bayesian networks and medical language modules to identify the optimal management strategy for laryngeal cancer. Bayesian networks and other modeling approaches can estimate and infer unobserved but relevant variables, which is advantageous in representing complex diseases. Natural language processing is becoming an increasingly common tool. Hong et al [27], Semenov et al [20,33], and Whitaker et al [38] used semantic tags, rules-based extraction, and the scispaCy-based natural language processing pipeline to extract their concepts, though these methods require arduous labeling—the process of manually highlighting terms and classifying them—and lack validation on external data sets. Vocabulary and expressions often differ outside of the training context, requiring developers to further refine their language models after release by using test data and real-life examples.

Cloud-based solutions have made it possible to process large-scale and heterogeneous data and push the boundaries of CDSSs to encompass broader scenarios. El-Sappagh et al [28] developed a mobile app that integrates data from wearable monitors (eg, vital signs, physical activity, and blood glucose levels) with the EHR to provide recommendations for managing type 1 diabetes mellitus. The system delivers spoken education and lifestyle recommendations to patients’ mobile devices, using an ontology generated from clinical practice guidelines, expert opinions, and other published sources. Meanwhile, in countries with nationally integrated health systems, citizens may be able to assemble their data across different institutions by using a secure server, such as Azure [31]. Henry et al [18] created a real-time prediction system for critically ill patients that alerts staff to elevated sepsis risk and tracks trends in vitals by using cloud-based technology. In the outpatient setting, Kawamoto et al [30] incorporated data from several EHRs into an existing risk prediction model.

A total of 3 studies described visualization tools. Gordon et al [29] generated visual aids to show patients’ thrombocytopenia trends, along with computer-generated calculated panel reactive antibody levels, to facilitate the judicious use of platelet transfusions by physicians and blood banks, and Thayer et al [36] used translated FHIR concepts to graphically display a patient’s asthma history within a chart. Xiao et al [41] were able to use knowledge graph ontologies to map FHIR and Observational Medical Outcomes Partnership (OMOP) data standards.

Despite the considerable benefits of cloud-based systems, they can present additional security challenges. These range from traditional cybersecurity problems (including problems related to data security, access control, and the transmission of data over a network) to more CDSS-specific concerns (such as privacy leakage, whereby models can be queried by outside parties). HL7 FHIR has put forward specific security protocols in response to safety concerns, including the use of secure http communication channels, open authorization, and provenance (documentation of the origin, possession, and history of a piece of data) techniques, among others [42].

### Data Management and Analytic Platforms

The rise in computing power and distributed system technologies facilitates general-purpose platforms that provide data standardization, data analysis, and model integration. Of the 39 included articles, 10 described FHIR-compliant data management and analytic platforms, as listed in Table 2. Although CDSSs require interoperability and multicenter clinical implementation, many clinical platforms did not support the real-time data integration that is necessary for clinical adoption.

**Table 2.** Summary of interoperable data management and analytic platforms.

Source, year	Functionalities	Strengths	Limitations
Gruendner et al [14], 2019	Data analysis and model deployment in clinical environments	Applied Docker virtualization that facilitates deployment across different environments	Poor performance on Extract, Transform, Load processing; relatively inefficient (bottleneck) FHIR^a^ transformation; and does not support real-time data processing
Haarbrandt et al [24], 2018	Integrating and transforming health data for oncology, cardiology, and infection control	Open-source platform that allows for patient-level data sharing	Does not support real-time data processing
Helm et al [43], 2022	Builds interoperability between FHIR and BPMN^b^	Supports BPMN clinical process models and improves explainability	Lacks some functionalities of the systems when used independently
Khalilia et al [25], 2015	Clinical predictive modeling using web services via HL7^c^ FHIR standards	Maintains good performance across many different algorithms	Does not support real-time data processing
Kopanitsa [44], 2019	Connects multiple health data systems	Has clear, effective workflows	Does not support real-time data processing
Marteau et al [45], 2022	Increases availability of clinical pediatric data using OMOP^d^ on FHIR	Implementation across multiple local environments	Not yet tested on real-word applications
Metke-Jimenez et al [46], 2018	Data searching, upgrading, and analyzing within multiple concept and category maps.	Syndication models automatically update the data	Does not support real-time data processing
Semenov et al [47], 2019	Clinical predictive analytics with text outputs to physicians and patients	Produces free-text outputs and graph visualizations pertaining to model recommendations	Limited support for real-time data processing.
Thiess et al [17], 2022	Application for support of shared decision-making in context of drug-drug interactions	Embedded interoperability functions within modular CDSS^e^ architectures	Performance testing limited to electronic health record training module
Xiao et al [41], 2022	Enables FHIR and OMOP interoperability with generated clinical knowledge graphs	Semantic foundation for development of explainable tools	Future iterations will require expansion of mapping systems

a FHIR: Fast Healthcare Interoperability Resources.

b BPMN: Business Process Model and Notation 2.0.

c HL7: Health Level 7.

d OMOP: Observational Medical Outcomes Partnership.

e CDSS: clinical decision support system.

Several papers addressed the challenge of integrating data from heterogeneous sources. Haarbrandt et al [24] proposed a platform that addresses this problem by developing techniques for converting disparate sources to FHIR standards prior to integration. The system is protected via fine-grained use-and-access control mechanisms that ensure secure data transmission among participating data sources. Metke-Jimenez et al [46] proposed an alternative approach to integrating several ontologies into a single web ontology language, allowing for updates to the ontology without changing the underlying data. For example, one could update the definition of *sepsis* and readily find all patients meeting the new definition. Distributed processing systems can be further enhanced via compartmentalization. Kopanitsa [44] and Semenov et al [47] developed a microservice platform that connects multiple systems via FHIR APIs. This platform was used to successfully deploy 400 CDSS models and 128 Bayesian diagnostic models in real time. Important to precision medicine, genomics data can now be linked to FHIR clinical data; 2 groups have created interoperability between the Variant Call Format for next-generation sequencing and FHIR [13,14].

Clinical information systems can aid in medical research, if properly designed. Although a prototype system proposed by Khalilia et al [25] ran 9 different machine learning models to generate data-driven, patient-level predictions, it lacked a researcher interface for the development and training of new models. In contrast, the KETOS platform proposed by Gruendner et al [14] allows researchers to request data sets, define cohorts, develop models, and deploy them as a web service. Both systems use Extract, Transform, Load pipelines to convert EHR data from their native format to the OMOP common data model format before storage. The KETOS platform’s comprehensive approach to data management and model deployment can aid researchers with limited backgrounds in data science.

### Auxiliary Modules and APIs

Artificial intelligence clinical information systems depend on robust and secure APIs to interact with the clinical environment. APIs define quality and security standards for each type of interaction with external systems (eg, EHR systems, web browsers, and medical devices). Article summaries are shown in Table 3.

**Table 3.** Summary of auxiliary modules and application programming interfaces (APIs).

Source, year	Functionalities	Strengths	Limitations
Altamimi [23], 2016	Provide security for FHIR^a^ functions to ensure patients’ privacy	Policies can be adjusted for circumstances (eg, emergency medical conditions can override privacy constructs)	There is no description of a user-side module, which would be necessary for clinical application
Alterovitz et al [48], 2015	Link clinical and genomic data with an FHIR-compliant API for clinical decision support	Ensures consistent semantics in clinical data and handles multiple types of genomic data	Effects of clinical decision support apps on decision-making and outcomes were not reported.
Dolin et al [49], 2021	Variant Call Format–to–FHIR genomic standard converter	Readily deployable to CDSS^b^	Limited independent data analysis and does not support real-time data processing
Kasparick et al [50], 2019	Model an FHIR-compliant protocol for artificial intelligence–based systems	Supports multiple devices and multiple domains of data	No clinical testing
Kopanitsa and Ivanov [51], 2018	FHIR-compliant APIs for data modeling	High data exchanging efficiency	No clinical testing
Gabetta et al [52], 2021	FHIR-on-OMOP^c^ platform to support data storage and retrieval	Use of standard OMOP vocabularies	No clinical testing
Guinez-Molinos et al [53], 2021	Reports COVID-19 test results to central authority	Interoperable and portable; functionally verified with a pilot study	Developed using a predecessor system
Mandel et al [26], 2016	Updating an API platform with FHIR standards	Improves API interoperability	Establishes feasibility, but effects on clinical decision-making and outcomes are unknown.
Rafee et al [54], 2022	LOINC^d^-mapped core data set for eligibility screening	Rapid EHR^e^ screening for patient recruitment	Relied on expert labeling, which limits scalability
Wood et al [55], 2021	Allows sharing of patient data among care provision sites for hematologic disorders	Compatible across EHRs	Framework alone; awaiting evidence of implementation
Yoo et al [56], 2022	Method for integrating CDSS applications with EHR	Transformation of EHR data into FHIR format for input into a reasoning engine	No validation of performance indices and usability of tested models

a FHIR: Fast Healthcare Interoperability Resources.

b CDSS: clinical decision support system.

c OMOP: Observational Medical Outcomes Partnership.

d LOINC: Logical Observation Identifiers Names and Codes.

e EHR: electronic health record.

Of the included articles, 5 described auxiliary modules and APIs. Mandel et al [26] applied FHIR standards to the SMART platform, improving its interoperability by providing standard authentication, authorization, and profiling. The prototype genomics standard developed by Alterovitz et al [48], meanwhile, is currently in trial use to facilitate the consistent integration of clinical and genomic information through SMART-on-FHIR application. The application developers found the FHIR v4.0.1 specification easy to leverage, even without prior experience with FHIR.

Although FHIR has predefined resources and mechanisms for transmitting orders and values, methods for creating and validating orders are not predefined. To address this issue, Kopanitsa and Ivanov [51] proposed an FHIR-based mechanism for integrating laboratory and hospital information systems. The system generated laboratory orders, using the available tests in the laboratory information system, and prompted the user for relevant information (such as how many laboratory samples should be collected and when they should be collected). It is challenging to make clinical information systems both highly interoperable and secure without compromising data workflows. SecFHIR is an XML-based security approach to FHIR resources. Using schema permissions built into XML documents, Altamimi [23] generated robust security profiles that were context-aware (eg, privacy constraints can be overridden in emergency care situations).

Timely data availability is another barrier to implementing CDSSs in high-acuity environments. Kasparick et al [50] proposed a reference model to address the timeliness challenge by connecting medical devices to FHIR servers. This approach allows the APIs to function as data sources for predictive analytic and decision support systems. By using these methods, clinical information systems can maintain high interoperability and security without compromising data workflow. This has allowed for the development of disease-specific data hubs, which facilitate research on rare conditions or for reporting the results of COVID-19 polymerase chain reaction tests from disparate testing sites to a central authority [53,55]. CDS hooks are another technology that permit the integration of EHR data into external health care applications [57]. Used in collaboration with SMART on FHIR, CDS hooks are triggered by a specific action within the EHR (ie, ordering a medication). The CDS hooks then link the corresponding EHR data to an environment of decision support applications [58]. These CDS applications can then push recommendations in the form of “CDS cards” to the clinician. These technologies are currently being tested in real-word settings [59,60].

## Discussion

### Key Findings

**Figure 1.**
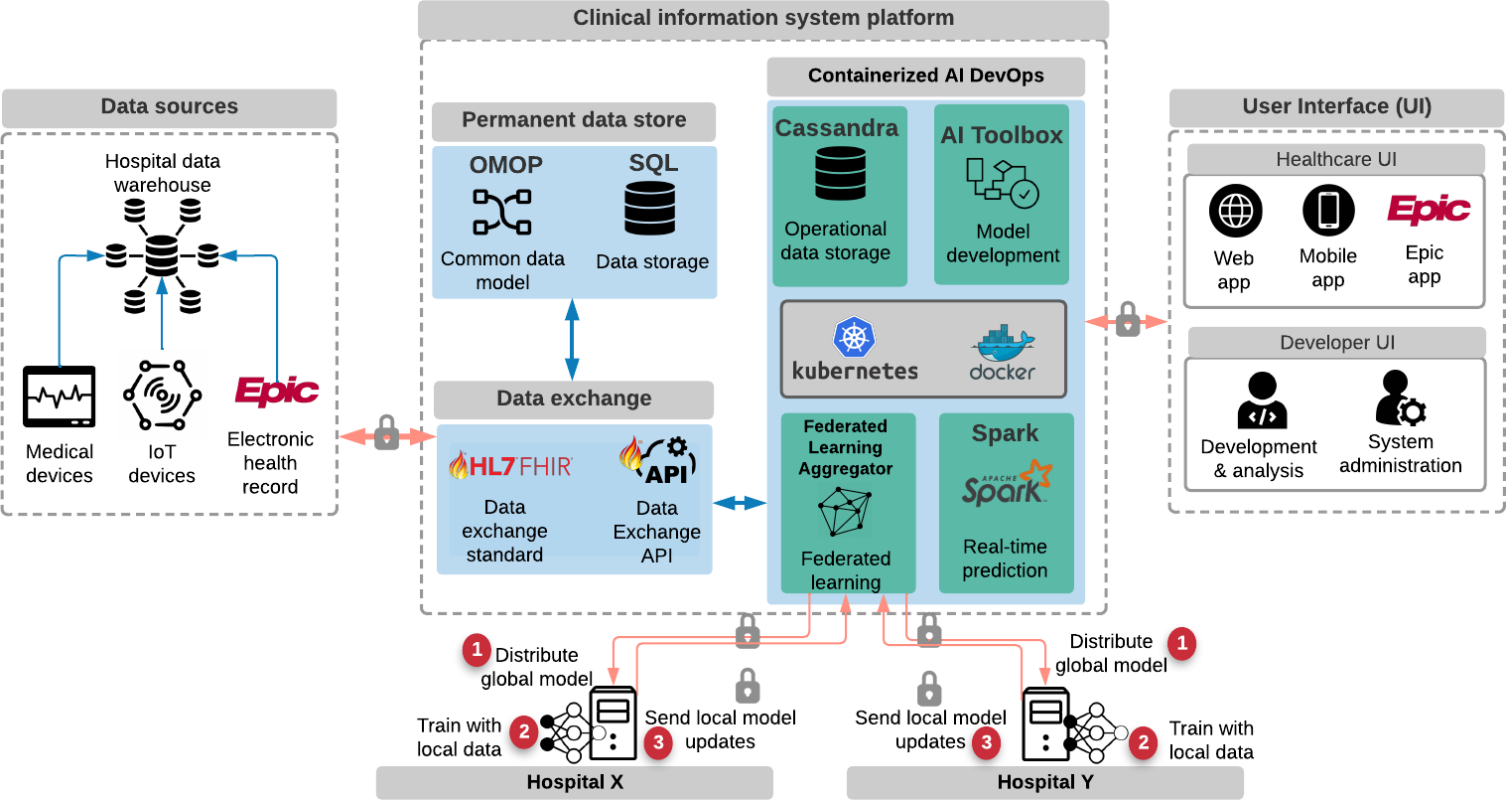
Sample model of a proposed machine learning–enabled clinical information system using FHIR data standards. AI: artificial intelligence; API: application programming interface; FHIR: Fast Healthcare Interoperability Resources; HL7: Health Level 7; IoT: Internet of Things; OMOP: Observational Medical Outcomes Partnership.

Although significant progress has been made in the field of FHIR data standards, this scoping review demonstrates that most CDSSs lack interoperability and actionable content. Several modules and APIs demonstrate the potential to enhance these systems, but they were not comprehensively integrated into the existing clinical workflows or were not validated on external patient populations. These limitations collectively reveal several opportunities to improve on existing methods to produce ideal clinical information systems, as illustrated in Figure 1.

### Foundational Infrastructures Tailored to Individual Needs

Ideally, clinical information systems would function as innovation hubs for patient care and health care research. Due to the proprietary nature of hardware and software systems in institutions, infrastructure components (eg, data transformation, model development, authentication, and monitoring) are often painstakingly created de novo. Platforms, such as KETOS, however, can enable the sharing of core infrastructure, greatly accelerating the development and deployment of applications that are tailored to the needs of individual researchers, groups, and projects [14]. This “health care application development hub” would be shared for different applications to reuse models and for data processing and analyzing services.

### Facilitating Interoperability Among Data Systems

Interoperability represents the goal of successful, cross-institutional sharing of data without additional, special effort. This remains in contrast to the current environment of fragmented data systems. Several elements of the current system impede progress toward integration and should be addressed. Sources of patient health information are numerous. At the point of data collection, clinicians may opt to store information in separate departments, erroneously duplicate patient descriptors, preferentially format or describe data, or use older data standards (HL7 v2 and v3). Further complexity may arise from the use of siloed systems, as exemplified by legacy systems built with local, stand-alone data conventions and incompatible ontologies [61]. The road to full interoperability is therefore paved with standards built to define, represent, transfer, and protect data as they travel between actors. Common communications standards, such as FHIR, have provided a useful framework for standardizing data transmission while maintaining semantic integrity at the patient level [15,56]. Importantly, these standards are built to mobilize data from legacy systems, making closely held data more publicly available [17]. By using OMOP-on-FHIR algorithms, pediatric data from Shriners Hospitals for Children can now be shared more widely by researchers [45]. More recently, 2 studies have examined the use of deep learning and transformer techniques to convert data elements in the EHR to interoperable FHIR standards, with subsequent application in prediction models [21,62]. Automation in data capture has the potential to reduce the costs and time associated with manual extraction.

### Overcoming Organizational Resistance to Interoperability Standards

Despite the benefits of an interoperable health data ecosystem, stakeholders are rarely incentivized to implement data standards. Organizational resistance to interoperability may stem from cultural differences, unfamiliarity with new technologies, or the fear that a newly adopted information-sharing standard may quickly become obsolete [63,64]. Among organizations, concerns regarding the loss of autonomy, a lack of trust, and the failure to realize financial gains impede interoperability and lead to so-called “information blocking.” The policies contained within the 21st Century Cures Act aim to improve information flow among actors in the system [65,66]. Apple, Google, and Samsung now have patient-facing health records that were developed along with FHIR standards to comply with these policies. In addition, while implementation models exist to help streamline the adoption of CDSSs, they contain important methodical flaws [67].

### Hiring Specialists to Manage Standards Adoption

Unfamiliarity with interoperability standards may represent a substantial hurdle to adoption and subsequent interoperability. This challenge creates demand for subject matter experts who are familiar with the architecture, function, and implementation of data standards. Such experts must be able to anticipate the specific challenges of adapting their particular legacy systems to the interoperable standard but also recognize the benefit of successful adoption to guide organizational buy-in [68].

### Timely Data Acquisition

The need for timeliness in data sharing is driven both by data availability and by opportunities for real-time treatment support. An obvious example of this can be seen with continuous glucose monitoring units for patients with diabetes, which provide a regular source of data that can be implemented immediately to adjust insulin therapy [69,70].

System scalability is also essential to this task. Many of the systems evaluated in this review cannot scale in real time, as data volume or velocity increases dynamically (eg, processing 1000 patients in real time vs processing 100 patients in a static, retrospective training cohort). When scalability is impaired, predictions may not be delivered in time to augment clinical decision-making. Health Insurance Portability and Accountability Act (HIPAA)–compliant cloud platforms can scale allocated resources on demand. Therefore, optimal clinical information systems must offer scalability that is commensurate with the expected volume and velocity of data.

### Minimizing Discoverable Patient Data

Each institution has policies that comply with municipal and federal security and privacy laws, making it challenging to share and aggregate data across multiple institutions. These challenges have been met with creative methods for aggregating multicenter data while maintaining patient privacy. One such method is to request only the minimum necessary information. This approach is emphasized heavily in the HIPAA and exemplified by El-Sappagh et al [28], who described a system that requests only the required EHR data elements for a specific patient. Other such mechanisms include authorization programs (enables specialized control over access to patient data), https, and WebSockets (Internet Engineering Task Force; provides secure communication over networks).

Alternatively, models can benefit from the knowledge derived from other data sets—usually in the form of model gradients or coefficients—without sharing the underlying data. This is known as *federated learning*—a system that trains on many local models with the same architecture and then aggregates the knowledge derived from each center into a global model (Figure 1). Although such an approach greatly reduces security and privacy risks by keeping the source records under the control of each local institution, even the gradients themselves pose a minor risk due to privacy leakage [28,71-75]. This risk, however, can be further reduced via the automated obfuscation of high-risk records or by adding noise to the gradients and coefficients before transmitting them to the central model. Given these advantages, federated learning is poised to supplant other methods for ensuring the data security and privacy of clinical information systems.

Finally, the recent explosion of large language models has raised further concerns regarding data privacy, as they are trained on clinical notes. This is an active field of study with multiple avenues for further research [76,77].

### Conclusions

Machine learning–enabled clinical analytic and decision support systems have the potential to improve health care by automating standardized workflows and augmenting clinical decision-making. Nevertheless, most CDSSs lack interoperability and evidence of clinical utility. Common data models and interoperable data management platforms can address these limitations, but most intelligent clinical platforms are also compromised by the inadequate scalability for supporting real-time data processing. Existing clinical information systems could be improved by using foundational code infrastructures, common data models, and secure data processing and analytics on real-time platforms. Further progress in implementing these elements can generate information systems that improve care by helping patients, caregivers, and clinicians make effective, well-informed clinical decisions.

## Supplementary material

10.2196/48297 Multimedia Appendix 1 PRISMA (Preferred Reporting Items for Systematic Reviews and Meta-Analyses) checklist for scoping reviews.

10.2196/48297 Multimedia Appendix 2 Search criteria.

## Abbreviations

API: application programming interface
CDS: clinical decision support
CDSS: clinical decision support system
EHR: electronic health record
FHIR: Fast Healthcare Interoperability Resources
HIPAA: Health Insurance Portability and Accountability Act
HL7: Health Level 7
LOINC: Logical Observation Identifiers Names and Codes
mCODE: minimal Common Oncology Data Elements
ML-CIS: machine learning–enabled clinical information system
NCIT: National Cancer Institute Thesaurus
OMOP: Observational Medical Outcomes Partnership
PRISMA-ScR: Preferred Reporting Items for Systematic Reviews and Meta-Analyses extension for Scoping Reviews
SMART: Substitutable Medical Applications and Reusable Technologies
SNOMED CT: Systematized Nomenclature of Medicine-Clinical Terms
