# Supplementary material for: Machine Learning–Enabled Clinical Information Systems Using Fast Healthcare Interoperability Resources Data Standards: Scoping Review
Source: JMIR Med Inform. 2023 Aug 24;11:e48297. doi: 10.2196/48297 (PMC10468818; doi:10.2196/48297)
Supplement: Multimedia Appendix 2 [file medinform-v11-e48297-s002.docx]

**PubMed:**

("fhir"[tiab])

AND

(“system”[tiab] OR "Online Systems"[Mesh] OR "Computer Systems"[Mesh])

AND

((“prediction”[tiab] OR “predictive”[tiab] OR “model”[tiab] OR "Forecasting"[Mesh] OR "Models, Theoretical"[Mesh])

OR

(“clinical decision-support”[tiab] OR “CDS”[tiab] OR “CDSS”[tiab] OR “clinical decision support”[tiab] OR "Clinical Decision Rules"[Mesh] OR "Decision Support Techniques"[Mesh]))

AND

("loattrfull text"[sb] AND English[lang])

Results: 26 articles

---------------------------------------------------------------------------------------------------------------------

**Embase:**

'fhir'

AND

('system' OR 'online system'/exp OR 'online system')

AND

('clinical decision support system'/exp OR 'clinical decision support'/exp OR 'clinical decision support' OR 'prediction and forecasting'/exp OR 'prediction and forecasting' OR 'prediction' OR 'model'/exp OR 'model')

AND

[english]/lim

Results: 45 articles (PubMed, Embase, and MEDLINE)

---------------------------------------------------------------------------------------------------------------------

**Web of Science:**

TS=(('fhir')

AND

('system')

AND

(clinical decision support' OR 'clinical decision support'/exp OR 'clinical decision support' OR 'prediction' OR 'forecasting' OR 'model'))

Results: 29 articles
